# Extraskeletal mesenchymal chondrosarcoma arising in adductor magnus with metastatic foci

**DOI:** 10.1259/bjrcr.20150117

**Published:** 2015-10-20

**Authors:** Kyle Hunter, Alan Alexander, Stephen Passerini, Allen Rovner, Ankur Garg

**Affiliations:** Department of Radiology, Aultman Hospital, Canton, OH, USA

## Abstract

Mesenchymal chondrosarcoma is a rare and aggressive chondrogenic neoplasm arising from the bone or the soft tissue. Mesenchymal chondrosarcomas develop outside the osseous structures in about one-third of cases, and the majority of these occur in the meninges and the brain parenchyma. Intramuscular extraskeletal mesenchymal chondrosarcoma (EMC) is exceedingly rare, with very few cases reported in the literature. Although mesenchymal chondrosarcoma has a high potential for metastasis, there have been no reports of pulmonary metastasis from an EMC of intramuscular origin. Here, we describe a patient who came to our facility with a history of progressively worsening left lower extremity pain and swelling, and was found to have pathology-proven EMC originating in the left adductor magnus, with complete workup demonstrating multiple bilateral pulmonary metastases in addition to a possible metastatic focus in the right adrenal gland discovered during the interval surveillance period.

## Introduction

First described in 1959 by Lichtenstein and Bernstein,^1^ mesenchymal chondrosarcoma is a rare form of high-grade mixed mesenchymal and cartilaginous malignancy arising from neural crest derivatives in the bone or the soft tissue.^2^ While chondrosarcoma is the third most common malignant bone tumour overall, mesenchymal chondrosarcoma comprises only 1% of all chondrosarcoma cases, with a frequency of 0.2–0.7 cases per 100,000.^2^ Approximately one-third of these lesions originate outside the osseous structures and are called extraskeletal mesenchymal chondrosarcomas (EMCs).^3^ Owing to its low incidence and variable length of disease-free survival, the natural history of mesenchymal chondrosarcoma is not fully understood. In the largest clinicopathological study of EMCs, which included 111 cases, it was found that this tumour is more aggressive than other types of chondrosarcomas, with a high incidence of distant metastasis.^4^

When compared with conventional chondrosarcoma, EMC differs in both sex predominance and the site of origin. Skeletal and extraskeletal conventional chondrosarcomas show a male predilection and typically arise in the pelvis and femur. There is a slight female preponderance in EMC, and a significant proportion of these develop in the central nervous system, meninges, maxillary sinuses, thyroid and soft tissues of the face.^3^ Very few EMC cases with primary intramuscular origins have been documented.^2,3^ EMC shows bimodal peak age incidences among adults, and these peaks correspond with predominant sites of origin. EMC infiltration of the central nervous system peaks at a mean age of 23.5 years (range: 5–48 years), whereas soft tissue and/or muscular involvement peaks, on average, at a mean age of 43.9 years (range: 21–62 years).^3^

### Cytogenetic and histopathological diagnosis

While the exact aetiology of EMC is not clear, cytogenetic analyses have elucidated that its tumorigenicity may be attributed to an identical Robertsonian translocation involving chromosomes 13 and 21 [der (13;21)(q10;q10)]. There have also been studies that suggest similarity of EMC pathogenesis to Ewing’s sarcoma, including a reciprocal translocation (11;22)(q24;q12) and trisomy 8. Furthermore, one case of mesenchymal chondrosarcoma demonstrated extra unidentifiable material on chromosome 22 with a breakpoint at 22q13, which is close to the breakpoint found in Ewing’s sarcoma at 22q12.^2^ EMC has a biphasic histological appearance that comprises sheets of undifferentiated mesenchymal cells and islands of well-differentiated cartilage. The poorly differentiated mesenchymal portions include sheets of tightly packed small cells that vary in morphology from round or elliptical to short and spindular. The nuclei of mesenchymal tissue are hyperchromatic and the cytoplasm is scant and indistinct. Immature cartilaginous islands are often situated at the periphery of the small cells and represent a zone of transition from the undifferentiated mesenchymal tissue to the more distinct hyaline cartilage. Larger, more mature cartilaginous islands seen in histological samples of mesenchymal chondrosarcoma may appear calcified or ossified.^2,5,6^ Calcification is common in EMC, being present in two-thirds of patients, but is not extensive. Overall, a wide spectrum of variability in histological appearance may be observed in EMC.^5^

With regard to histopathological diagnosis of EMC, immunohistochemical analysis provides assistance in narrowing the differential diagnosis. Mesenchymal chondrosarcoma stains positively for S100 protein and is negative for the presence of cytokeratins (CKs), which are found in synovial sarcoma and spindle cell variants.^2^ The S100 protein, which is a commonly used marker for differentiating chondrogenic neoplastic tissue, is only positive within the cartilaginous islands in mesenchymal chondrosarcoma tissue samples. There is also partial positivity for vimentin, an intermediate filament protein expressed in mesenchymal cells, within the sheets of poorly differentiated small cells of mesenchymal chondrosarcoma.^2,3^ Delineation of mesenchymal chondrosarcoma from non-mesenchymal small cell cancers such as malignant lymphomas, metastatic carcinomas, neuroblastomas and embryonal rhabdomyosarcomas may be achieved with immunohistochemical markers such as common leukocyte antigen, CK, non-specific esterase and desmin, respectively.^3^ However, Ewing’s sarcoma, another entity in the differential diagnosis for mesenchymal chondrosarcoma, demonstrates CD99 expression, which is confounded by CD99 expression in mesenchymal chondrosarcoma as described by Müller et al.^7^

### Radiological features

Plain radiograph, while important as an initial step in the radiological evaluation of suspected EMC, often has limitations secondary to potential overlapping of the adjacent structures and lower density resolution. CT images of EMC eliminates the uncertainty as to whether the structures are overlapping or if there is contiguity with the bone, which would posit the more probable existence of conventional chondrosarcoma. In a study by Chen et al,^5^ CT scans of patients with EMC revealed a variety of calcification patterns among eight patients. One of the most valuable imaging findings related to EMC in this cohort was a ring-and-arc pattern of mineralization. In their study, patients with peripherally distributed EMC exhibited a wide and dense calcification, while there was only one case (one-third) of centrally distributed EMC that had focal calcification. Because the cohort was too small to analyze for any sensitivity and specificity of this imaging finding with any reasonable power, the diagnostic worth of calcification patterns in EMC requires further substantiation.

MRI of EMC yields a more extensive evaluation of soft tissue involvement. EMC has been demonstrated as isointense and hypointense signal on *T*_1_ and heterogeneously hyper- and hypo-intense signals on *T*_2_ weighted images. Intratumoral mineralized and non-calcified components of peripheral EMC exhibit low and high signal intensity on *T*_2_ weighted images, respectively. This finding of hyperintensities surrounding areas of hypointense signals is known as the “black pepper” sign and is considered to be one of the most important imaging characteristics of the disease. Diffuse heterogeneous or nodular enhancement can be found in both mineralized and non-mineralized areas. This is suggestive of the increased vascularity within the EMC tissue and is also an important diagnostic finding.^5^

### Treatment and prognosis

Owing to the rarity and the variable nature of the disease, treatment of mesenchymal chondrosarcoma is incompletely studied and EMC even less so. Surgery is the primary form of local therapy, with patients undergoing wide resection of the lesion demonstrating better survival. Currently, the National Comprehensive Cancer Network guidelines recommend that mesenchymal chondrosarcoma be treated with vincristine, doxorubicin and cyclophosphamide alternating with ifosfamide and etoposide. This is a treatment regimen used typically in cases of Ewing’s sarcoma. The efficacy of adjunctive radiation therapy is poorly defined, with purely anecdotal reports of its value.^2^

Prognosis of all cases of mesenchymal chondrosarcoma is widely variable, with 10-year overall survival rates ranging from 21% to 67%.^2^ It has been reported that some patients die shortly after diagnosis, while others may live for long periods even in the presence of metastatic disease. Decreased survival has been associated with haemangiopericytomatous features, but prognostic indicators are again largely conjectural, as no single adjuvant treatment protocol has been adopted.^2^

## Case report

**Figure 1.**
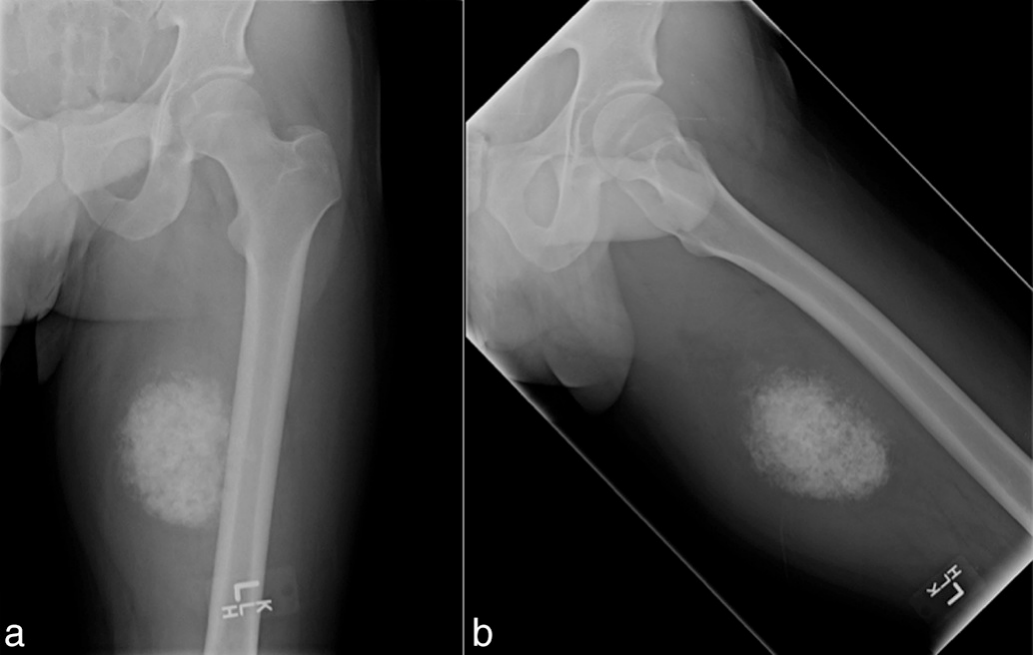
(a,b) Anteroposterior and lateral (frog leg) radiographs of the left femur demonstrate an extraskeletal lesion with dense minerlization, adjacent to the medial aspect of the femur.

**Figure 2.**
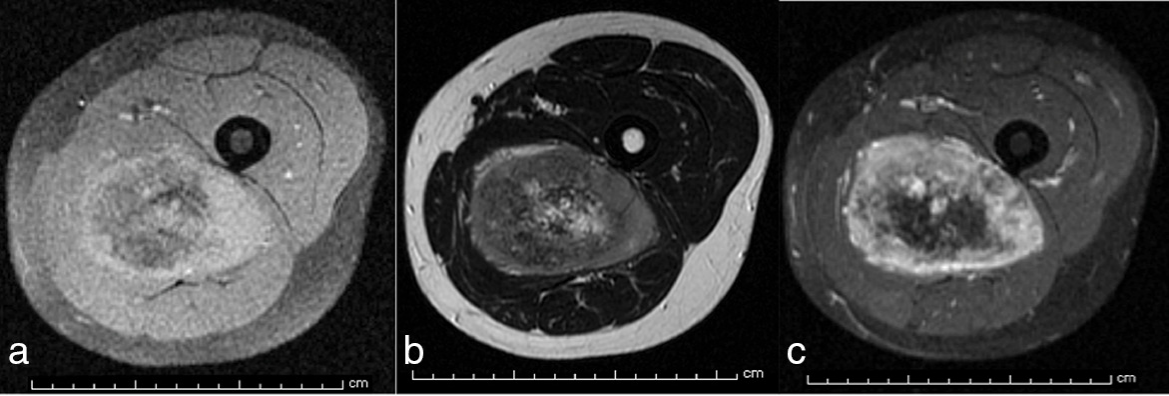
(a–c) Axial MRI sequences (*T*_1_ fat-saturated, *T*_2_ and *T*_1 _post-contrast fat-saturated) demonstrate a large mass centred in the adductor magnus of the left thigh with internal calcification and peripheral vascularity. The mass demonstrates intermediate *T*_1_ signal and increased heterogeneous *T*_2_ signal (black pepper sign) compared with background muscle. There are no signal abnormalities within the adjacent femur to suggest bony invasion.

**Figure 3.**
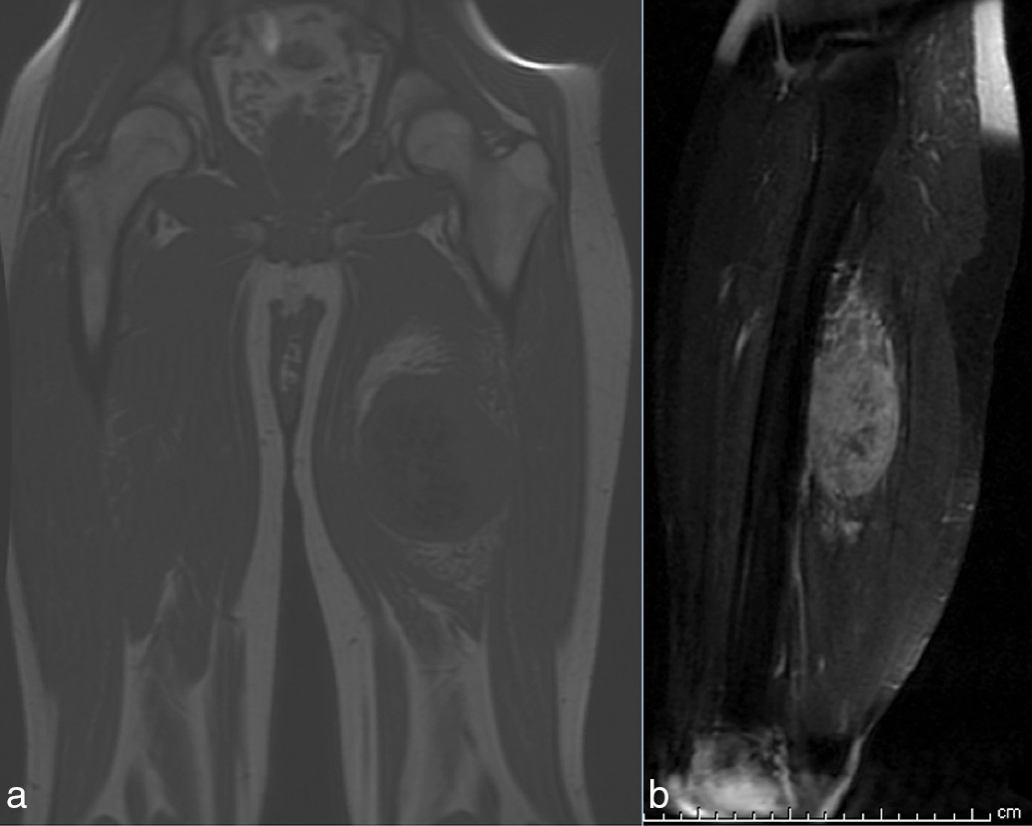
(a,b) Coronal *T*_1_ and sagittal short tau inversion-recovery images demonstrate the superior and inferior margins of the mass, again without evidence of signal abnormality in the adjacent femur to suggest bony invasion.

A 32-year-old male with no significant past medical history presented to our facility with a history of worsening pain and swelling in the medial aspect of his proximal left lower extremity over the past several years. Anteroposterior (AP) and “frog leg” lateral radiographs of the left lower extremity revealed a dense lesion consistent with dense matrix, adjacent to the medial aspect of the femur (Figure 1a,b). An MRI of the left lower extremity revealed that the lesion was of extraskeletal origin, arising from the adductor magnus, without any involvement of the femur (Figures 2a,b, 3). The solid heterogeneous mass was located in the posterior compartment of the proximal-to-mid thigh and measured 9.6 × 7.3 × 13 cm in the transverse, AP and vertical dimensions, respectively. Multiple tiny hypointense foci, which corresponded to the calcific density noted on the prior plain radiographical studies, were noted centrally. The mass was otherwise of intermediate soft-tissue intensity on *T*_1_ weighted images and moderately hyperintense to muscle on *T*_2_ weighted images. Following intravenous administration of gadolinium, moderate enhancement of the mass, excluding the central portion, was observed. Superiorly and inferiorly, the margins of the mass were indefinite, and the mass demonstrated peripheral vasculature, which was most prominent at the cephalad and caudad margins. The mass was centred within the adductor magnus muscle fibres that were displaced around the mass. Anterolaterally, the mass was very closely approximated to the posterior cortex of the femur and the fascial margins that separated the quadriceps and the posterior compartments (Supplementary videos). No obvious signal abnormality was noted within the femur to indicate invasion or primary osseous origin. Post-excision pathology confirmed the presence of mesenchymal chondrosarcoma.

**Figure 4.**
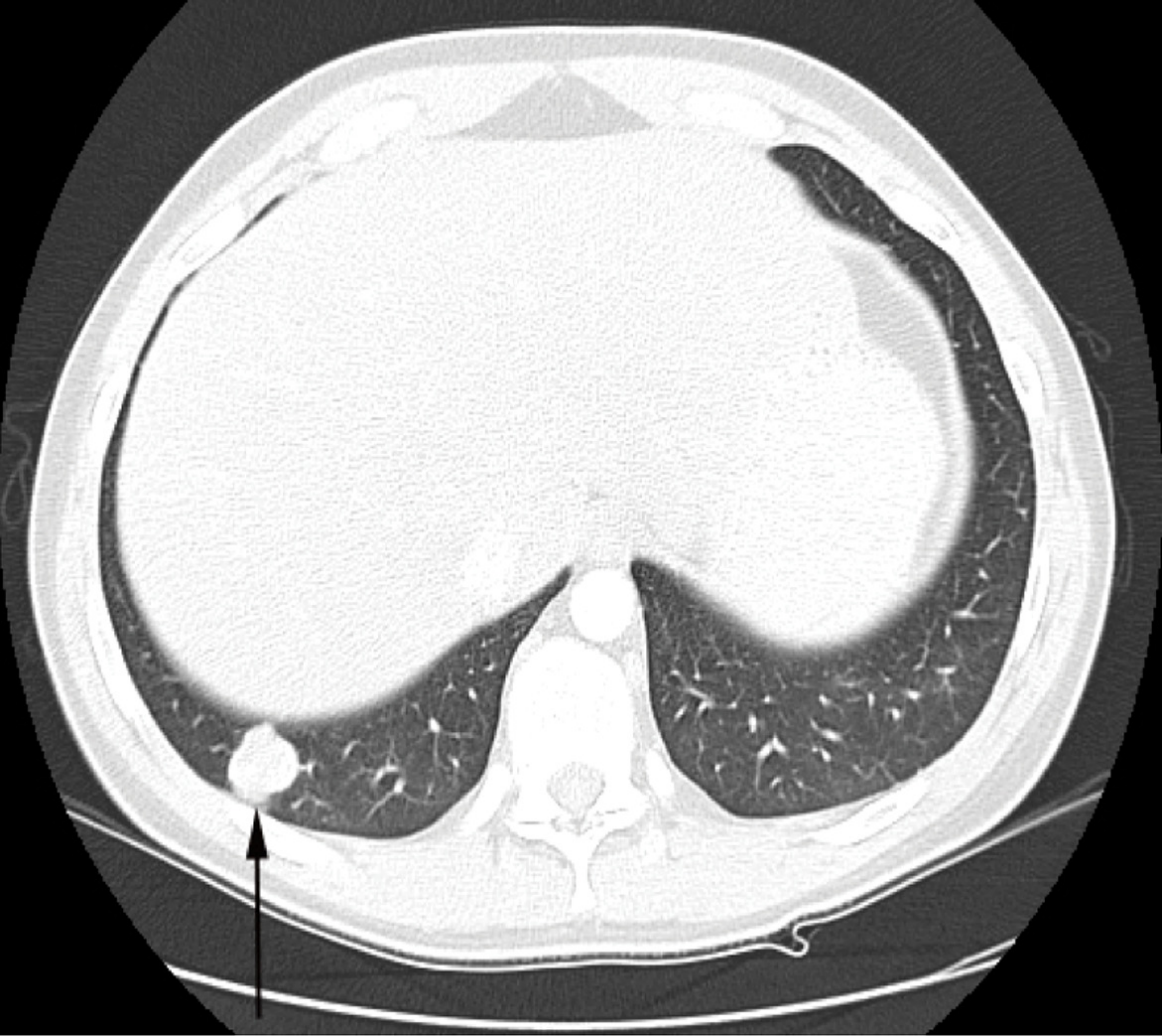
An axial CT image of the chest, with intravenous contrast, demonstrates a 2.3-cm right basilar nodule (arrow), confirmed to be a metastatic focus. Multiple additional smaller pulmonary nodules were seen throughout the lung parenchyma (not shown).

**Figure 5.**
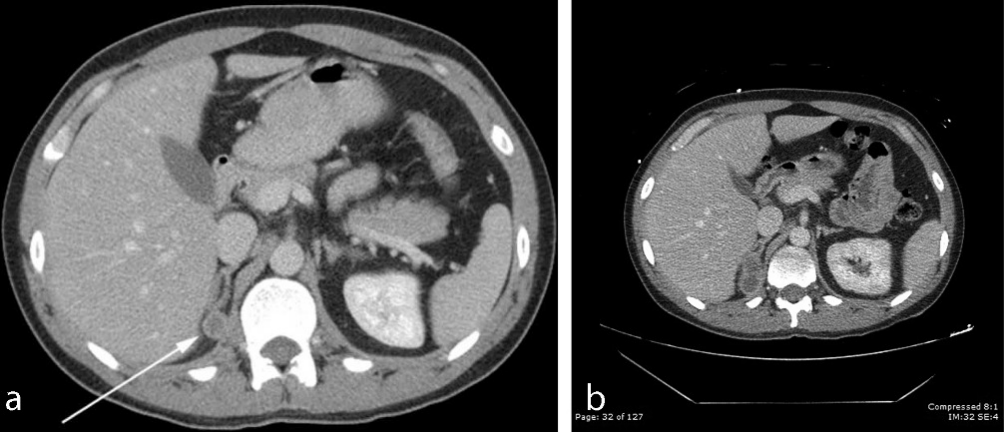
(a,b) A hypodense pedunculated mass measuring 1.6 cm in greatest dimension, representing adrenal metastasis (arrow).

In the interval, surveillance imaging for metastatic disease with CT scan of the chest, abdomen and pelvis was performed. At 18 months, multiple bilateral non-calcified pulmonary nodules concerning for metastasis were identified (Figure 4). The most prominent pulmonary nodule was present within the posterior right base, measuring 2.3 cm in the greatest dimension and abutting the pleura (Figure 5). With regard to the abdominal and pelvic series, a new right adrenal lesion appearing as a hypodense pedunculated mass measuring 1.6 cm in the greatest dimension was noted. There was no evidence of osseous metastases.

## Discussion

This case of EMC highlights a rare tumour arising solely within the adductor magnus muscle. To date, there have been no documented cases of intramuscular EMC with metastatic foci within the lungs or adrenals. Given the low incidence of this malignancy, the literature is rather limited with regard to diagnostic approach, treatment and prognosis. Several radiological features have redemonstrated support for the diagnosis of EMC, although there are many shortcomings inherent to these diagnostic modalities. The densely calcified masses on plain radiograph with ring-and-arc calcification on CT scan and the “salt and pepper” sign on *T*_2_ weighted images in combination suggest the diagnosis, although all of these features need not be present, these features were present in the case at hand.

The road to a more definitive diagnosis is paved with wide excision of the mass as the initiation of therapy. The excised tumour may be assessed with a series of histopathological markers. EMC has shown S100, vimentin and CD99 positivity, along with the absence of CKs, but even these markers are not completely sensitive and may confound the differential diagnosis. Cytogenetic studies have begun to show a link with Ewing’s sarcoma, and genetic testing will likely become the diagnostic and therapy-guiding modality of choice. With rapid diagnosis, the uncertain natural course of the tumour may be altered by initiating therapy that could possibly prevent haematogenous spread as seen in our patient. Furthermore, more efficient diagnosis of this entity through radiological, histopathological and cytogenetic studies may extend survival among EMC patients, allowing for more tailored therapies to be developed.

## Learning points

1. EMCs are rare, malignant soft-tissue sarcomas that typically arise in young adults.
2. EMC has a predilection to arise in the meninges and spinal dura, but can also be found in the lower extremity.
3. On CT scan, EMC appears as a soft-tissue mass, isodense to muscle with heterogenous enhancement. Calcifications in a “ring-and-arc” configuration may be seen.
4. MR evaluation reveals isointensity to muscle on *T*_1_ weighted imaging. On *T*_2_ weighted imaging, heterogenous hyperintense and hypointense signal may be observed, reflecting intratumoral mineralized foci. This is known as the “salt and pepper” or “black pepper” sign.
5. The treatment of EMC includes wide local excision and chemotherapeutic agents used in the treatment of Ewing’s sarcoma.
6. The prognosis of EMC is variable, with 10-year survival ranging from 21% to 67%.
